# Dynamic quantification of canopy structure to characterize early plant vigour in wheat genotypes

**DOI:** 10.1093/jxb/erw227

**Published:** 2016-06-15

**Authors:** T. Duan, S.C. Chapman, E. Holland, G.J. Rebetzke, Y. Guo, B. Zheng

**Affiliations:** ^1^College of Resources and Environmental Sciences, China Agricultural University, Beijing 100193, China; ^2^CSIRO Agriculture, Queensland Biosciences Precinct, 306 Carmody Road, St Lucia, QLD 4067, Australia; ^3^CSIRO Agriculture, PO Box 1600, Canberra, ACT 2601, Australia

**Keywords:** 3D reconstruction, high-throughput phenotyping, image analysis, leaf structure, morphogenesis, segmentation.

## Abstract

Early vigour is an important physiological trait to improve establishment, water-use efficiency, and grain yield for wheat. Phenotyping large numbers of lines is challenging due to the fast growth and development of wheat seedlings. Here we developed a new photo-based workflow to monitor dynamically the growth and development of the wheat canopy of two wheat lines with a contrasting early vigour trait. Multiview images were taken using a ‘vegetation stress’ camera at 2 d intervals from emergence to the sixth leaf stage. Point clouds were extracted using the Multi-View Stereo and Structure From Motion (MVS-SFM) algorithm, and segmented into individual organs using the Octree method, with leaf midribs fitted using local polynomial function. Finally, phenotypic parameters were calculated from the reconstructed point cloud including: tiller and leaf number, plant height, Haun index, phyllochron, leaf length, angle, and leaf elongation rate. There was good agreement between the observed and estimated leaf length (RMSE=8.6mm, *R* ^2^=0.98, *n*=322) across both lines. Significant contrasts of phenotyping parameters were observed between the two lines and were consistent with manual observations. The early vigour line had fewer tillers (2.4±0.6) and larger leaves (308.0±38.4mm and 17.1±2.7mm for leaf length and width, respectively). While the phyllochron of both lines was quite similar, the non-vigorous line had a greater Haun index (more leaves on the main stem) on any date, as the vigorous line had slower development of its first two leaves. The workflow presented in this study provides an efficient method to phenotype individual plants using a low-cost camera (an RGB camera is also suitable) and could be applied in phenotyping for applications in both simulation modelling and breeding. The rapidity and accuracy of this novel method can characterize the results of specific selection criteria (e.g. width of leaf three, number of tillers, rate of leaf appearance) that have been or can now be utilized to breed for early leaf growth and tillering in wheat.

## Highlight

An efficient workflow based on photos to monitor early growth and development of isolated wheat plants dynamically is described

## Introduction

Vigorous growth in the early stage, affected by various genetic traits (Rebetzke and Richards, 1999; Rebetzke *et al.*, 2007), is an important physiological trait to contribute to improved water-use efficiency and grain yield of wheat (Botwright *et al.*, 2002). Early vigour for wheat can be increased through selection of larger grain size (embryo size) (White *et al.*, 2012), but is more commonly selected using leaf traits, such as the maximum width of the expanded third leaf, which was shown to be correlated to total leaf area of young seedlings (Zhang *et al.*, 2015).

In Australian wheat production systems, yield potential is limited by the plant-available soil water during the growing season (Richards *et al.*, 2002). Greater early vigour has been identified as a physiological trait to improve the water-use efficiency in the rain-fed environment (Siddique *et al.*, 1990; Richards, 1991). Wheat genotypes with early vigour traits should reduce soil evaporation through quicker shading of the soil surface in the early growth stage, and retaining more water that can be available to the roots for transpiration (Siddique *et al.*, 1990). In addition, a shaded soil surface would cool the crop canopy and reduce vapour pressure deficit within the canopy. Both mechanisms could improve transpiration efficiency (Fischer, 1979), with crop seasonal water-use efficiency increased by up to 25% (Regan *et al.*, 1992) when combined with improvement of economic yield (López-Castañeda and Richards, 1994). A further benefit of greater early vigour is to reduce the light availability at the soil surface and to suppress weed growth (Lemerle *et al.*, 1996; Coleman *et al.*, 2001). Quicker canopy growth soon after emergence also improves light interception potentially to enhance crop growth rate, biomass, and net grain yield (Regan *et al.*, 1997). Early vigour may be associated with both larger leaves and quicker ground cover of leaves and tillers (Mullan and Reynolds, 2010), which are partly determined by embryo size and environmental growth factors (e.g. temperature and soil moisture) (Mohsen *et al.*, 2011; Rebetzke *et al.*, 2014).

The traditional phenotyping technologies are time-consuming and expensive with anthropogenic interference (Richards and Lukacs, 2002; Bertin *et al.*, 2010). Breeders have to select new genotypes more efficiently for target population environments to secure food requirement in the future as it takes 7–15 years for a breeding cycle (Chapman *et al.*, 2012; Zheng *et al.*, 2013), and crop production must double by 2050 to meet the global population demand (Chilcoat, 2015). Consequently, there is now greater interest in developing rapid and non-destructive technologies for high-throughput phenotyping (e.g. Chapman *et al.*, 2014; Deery *et al.*, 2014). Parameters (e.g. leaf appearance rate, tillering rate) derived from such technologies can also be used in crop simulation models in order to simulate other phenotypes. However, measurement of these parameters is extremely time-consuming in dynamic plant experiments. Canopy reconstruction from multiview images provides an economical and non-destructive method to monitor plant growth and development dynamically, in order to characterize large numbers of breeding lines (Hartmann *et al.*, 2011; Paproki *et al.*, 2012). Many commercial methods use only a small number of views of the plant to estimate its size, and cannot be used to undertake detailed analysis, such as the LemnaTec Scanalyzer (LemnaTecAG, Aachen; www.lemnatec.de) (Junker *et al.*, 2015).

Plant architecture plays an important role to select advanced lines in breeding programmes (Schwartz *et al.*, 1987; Sakamoto and Matsuoka, 2004; Cabrera-Bosquet *et al.*, 2012). Several technologies have been well developed to obtain 3D spatial structures of crop canopies (Lou *et al.*, 2014; Pound *et al.*, 2014), such as LiDAR (Omasa *et al.*, 2007), time-of-flight laser (Kazmi *et al.*, 2014), and ultrasonic sensing (McCarthy *et al.*, 2010). The technologies are expensive, with limited operation environments. Multiview images can be used to reconstruct 3D canopies given recent advances in photogrammetry information computing (Quan *et al.*, 2006; Kumar *et al.*, 2012). Combined with pixel analysis of RGB images (Fang *et al.*, 2013) or high spatial resolution broadband imagery (Nigon *et al.*, 2013), crop phenotypes can be extracted from images (Clark *et al.*, 2011; Bucksch *et al.*, 2014; Klukas *et al.*, 2014; Zou *et al.*, 2014).

To date, imaged-based phenotyping has been used to analyse the complete structure of plants with wider leaves, such as leaf length, width, and stem height of *Gossypium hirsutum* (Paproki *et al.*, 2012). The algorithm is also suitable for broad cereal leaves to reconstruct a 3D point cloud and the leaf surface to estimate the phenotypic parameters related to growth (Fang *et al.*, 2013; Kempthorne *et al.*, 2014). However, this photogrammetry is not suitable for narrow- and thin-leaved plants, such as wheat and rice. Several studies have been conducted for 3D barley and wheat reconstruction using multiview images (Hartmann *et al.*, 2011), which presented a fully automatic approach to reconstruct the 3D meshes of leaves at mature stages. However, these studies are focused on the point cloud reconstruction (Kumar *et al.*, 2012) which is suitable for light distribution simulation, but cannot characterize other phenotypes related to leaf appearance, tillering, etc., which are more likely to be directly related to genetic control.

The aims of our study were to (i) develop an efficient phenotyping workflow to capture suitable image sets for two cultivars with contrasting early vigour traits; (ii) reconstruct the point cloud and segment it into individual organs; (iii) fit the leaf structure using the local polynomial regression algorithm; and (iv) monitor the dynamic growth development of wheat seedlings into the early tillering stage.

## Materials and methods

**Fig. 1.**
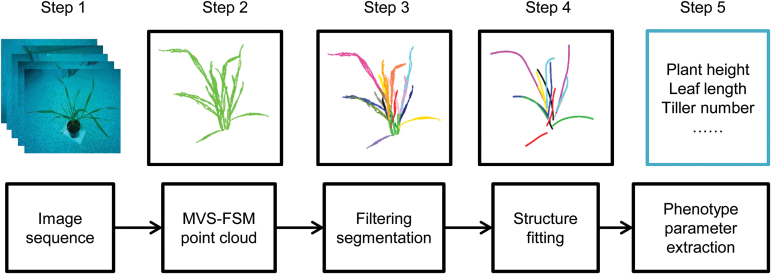
Pipeline of 3D structure fitting and phenotype parameter extraction based on the image set. The image set comprises images taken by one or more cameras around single plants at 60–70 positions, with reconstructed point clouds derived using the MVS-FSM (Multi-View Stereo and Structure From Motion) algorithm. See text for details. (This figure is available in colour at *JXB* online.)

This study involved five major steps to reconstruct the 3D structure of wheat seedling and extract phenotype based on the multiview images (Fig. 1): (i) capture the image set for an individual plant; (ii) reconstruct 3D point clouds using the VisualSFM (Wu, 2011), which is based on the MVS-SFM (Multi-View Stereo and Structure From Motion) algorithm; (iii) segment point clouds into individual organs; (iv) fit leaf midribs with local polynomial function; and (v) extract phenotype parameters from a dynamic monitoring of plant structure.

### Glasshouse experiments

An experiment was conducted in a naturally lit glasshouse at CSIRO in St Lucia, Brisbane (27.50°S, 153.01°E). Two wheat genotypes were selected with a contrasting early vigour trait (i.e. Cycle-6-1 and Lincoln). Lincoln is a common commercial variety while Cycle-6-1 is a new high early vigour S0-derived line from the sixth cycle of a broad-based recurrent selection programme built from 28 high vigour lines collected globally (Zhang *et al.*, 2015). Cycle 6-1 and Lincoln have similar phenology with similar flowering time. Cycle-6-1 has larger leaves when young compared with Lincoln. Single seeds were planted into pots (10cm high, 10cm diameter) after germination on 9 May 2015. Nine pots were accommodated into a block (plot) to form a ‘minicanopy’. Each genotype was replicated in three plots (27 pots total), with the six ‘plots’ of Lincoln and Cycle-6-1 being randomly distributed across a table in the glasshouse. All pots were randomly reordered within blocks every day to reduce the effects of uneven light distribution on plant growth and development, and blocks were also randomly ordered across and along the table. Plants were irrigated as required for normal pot experiments. Fertilizers were applied at sowing, and at 8 d and 19 d after sowing as per the recommendations of the growth requirement of Cycle-6-1 and Lincoln [i.e. dissolving 9g of fertilizer mix into 4.5 litres of water and applying over 2 m^2^ of pot area; fertilizer mix: analysis (% w/w), total nitrogen (N), 25.0; phosphorus (P) as water soluble, 5.0; potassium (K) as sulphate, 8.8]. In the glasshouse, the day and night temperatures were controlled at 22 °C and 15 °C, respectively, and relative humidity was 65%. The actual weather conditions were recorded in the glasshouse by a TinyTag data logger (Hastings Data Loggers, UK). The actual temperatures were 21.8±0.8 °C and 15.2±0.7 °C in the day and night, respectively. Daily maximum photosynthetic radiation was 805.2±161.0W m^−2^. The actual relative humidity was 65.8±10.5%.

### Capturing image sets for individual plants

**Fig. 2.**
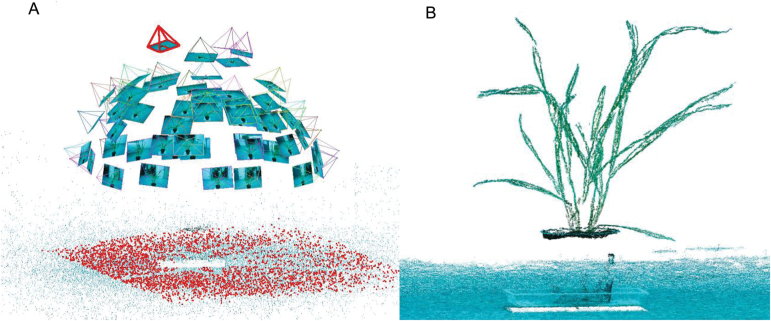
(A) Camera positions for the image set and (B) operation result of 3D point cloud using the VisualSFM system (a real data example: Lincoln, 5 June, six leaves on the main stem). The camera positions (vertexes of the cones) and directions (four edges of cones) were estimated for 61 photos by VisualSFM (A). The thick dots on the bottom in (A) indicate the designated shooting area of the selected camera (thick cones in A). The dense point clouds were generated after estimation of camera positions and directions (B). (This figure is available in colour at *JXB* online.)

On 16 May (7 d after sowing), one of the nine individual plants in each of six plots was randomly selected, with the plants at the first leaf stage for Cycle-6-1 and the second leaf stage for Lincoln on this day. The same six selected plants were used to take image sets at 2 d intervals from the first or second leaf stage (16 May) to the sixth leaf stage on the main stem for Cycle-6-1 (9 June) and Lincoln (5 June). Hence, each plant was imaged 13 and 11 times for Cycle-6-1 and Lincoln, respectively. Multiview image sets were taken from 60–70 positions using a Canon PowerShot ELPH 110 HS camera with a 24–120mm Equivalent UA Lens (http://www.usa.canon.com). This camera captures 16 mega pixels (4608×3456) and had its infra-red (IR) filter removed, replaced by a filter to capture near infra-red (NIR) wavelengths in the red band (http://www.maxmax.com/RemoteSensingcamerasi.htm). It has a strong NIR reflectivity and is more sensitive for the colour green, so it can also be called a ‘vegetation stress camera’. For an image set, photos were taken in a hemispherical distribution around the plant, with it positioned on a grey carpet in a clear room with uniform illumination conditions (Fig. 2A). Other backgrounds of photography environments were tested, such as high contrast poster and QR Code. However, the algorithm of point cloud reconstruction was not sensitive to background (data not presented). The intervals between camera positions were ~20–30 ° in the vertical and horizontal axes on the surface of a hemisphere. Several extra photos were taken directly above the plant, which were high enough to ensure a full view of the whole plant (Fig. 2A, positions marked as thick cones). In a preliminary experiment, other image acquisition methods were tested, namely rectangular movement trajectory of the camera position centring on the plant, the rectangular movement trajectory of the camera position parallel to the top of the plant, and the circular movement trajectory of the camera position around the plant side. The 3D reconstruction results showed that the hemispherical trajectory was the best option for wheat seedlings (data not shown). The lengths and maximum widths of all leaves on each of the six plants were manually measured using a ruler after imaging. The measured values were used to validate the accuracy of 3D reconstruction from image sets. We also tested all of our methods with a normal RGB camera of similar specification which worked just as well (data not shown). We continued with the ‘vegetation stress’ camera as we had also been considering looking at changes in NIR reflectance over time (data not presented here).

### Three-dimensional reconstruction from the multiview image sequence

The 3D reconstruction of wheat seedlings from an image set was undertaken using VisualSFM software, which is based on the MVS-SFM algorithm.

SFM included two main processes: (i) searching for the matching points from the image sequence of multiview through a Scale-Invariant Feature Transform (SIFT) keypoint detector and descriptor using the Approximate Nearest Neighbor (ANN) algorithm (Arya *et al.*, 1998); and (ii) calculating the corresponding co-ordinates of 3D key points and camera parameters of each image (position and direction). The SFM algorithm is based on the geometric principle of perspective projection (i.e. establishing the relationship between 2D and 3D points through perspective projection). For example, *X* is a 3D point and *x* is its corresponding 2D pixel on one of the images. The perspective projection matrix is *P:*

$$P\;=\;K(\frac{R}{t})\;$$ (1)

where *K* is the intrinsic parameter of the image including focal length, principal point offset, and axis skew, *R* is the direction of the camera, and *t* is the position of the camera. The projection equation is obtained by:

x=PX (2)

The SFM algorithm is used to extract the matching 2D points (*x*_1_, *x*_2_, …) from pairs of images in the full set. Then the 3D point co-ordinates are calculated with the corresponding 2D matching points and the projection equation. The sparse 3D point cloud and camera parameters of each image are indicated in Fig. 2A. After the camera parameters (position and direction for each image) were calculated in the SFM step, the MVS algorithm (an open source software PMVS; http://www.di.ens.fr/pmvs/) was used to calculate the dense 3D point cloud (Fig. 2B). The PMVS is a multiview stereo software that uses image sequence and camera parameters, then reconstructs 3D point clouds of the target (Fig. 2B). The point clouds were pointing to an arbitrary direction after reconstruction. For easier post-processing, the plants were moved and rotated into a standard co-ordinate system, which assumed the plant was grown from the origin and straight up (positive *z*-axis). The reconstructed 3D co-ordinates are unitless. For each reconstructed target scene presented here, the tray length (0.10 m) was used as a scale to reproject 3D co-ordinates into the real world.

### Point cloud segmentation

**Fig. 3.**
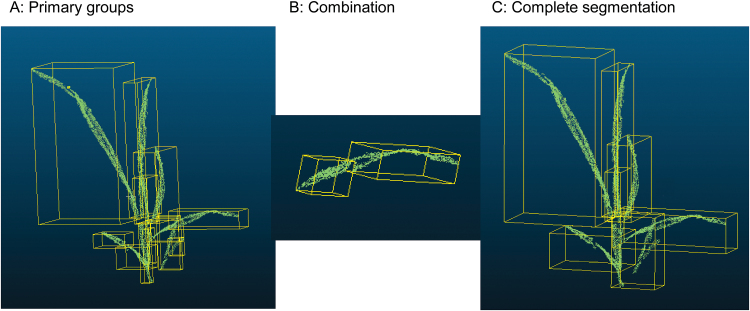
Processing of point cloud segmentation using the Octree model in CloudCompare (a real data example: Lincoln, 28 May, four leaves on the main stem). Octree level: 8 (grid step=0.0026243), min points per component: 10. (This figure is available in colour at *JXB* online.)

For each plant, the individual organs were segmented from the unordered point cloud using the Octree algorithm in CloudCompare (http://www.danielgm.net/cc/ ; 3D point cloud and mesh processing software). At each measurement date, the parameters of the Octree (Octree level indicates the minimum number of points per component) were adjusted to separate point clouds into several primary groups, which were typically slightly more than the number of individual organs (Fig. 3A). Then the primary groups were manually merged into individual organs depending on the topology relationship (Fig. 3B). The step of group merging was repeated until organs were segmented (Fig. 3C). The leaf ranks were identified according to positions (3D co-ordinates) in the adhered main stem and tillers. The tiller ranks were also identified according to positions (3D co-ordinates) and the associated leaf rank in the main stem. Due to self-shading among organs, it is hard to segment point clouds into individual organs and to identify the organ ranks after the sixth leaf stage (images were taken on further dates, but data are not shown here). This merging of some groups (Fig. 3B) is the only manual step in the workflow and takes ~1–15min depending on the organ number per image set. See Table 1 for the details of the main steps in the workflow to process the multiview images.

**Table 1.** Detailed description of the workflow to process multiview images into individual organs including the manual measurement for validation of estimated phenotyping The time requirements of manual measurements only include leaf width and length. The runtime of organ segmentation involves the computing time and manual interference.

Step	Image acquisition	3D reconstruction	Segmentation	Phenotyping extraction	Manual measurement time
Software/ equipment	Canon Power Shot	VisualSFM	CloudCompare	R	Ruler (length) Vernier caliper (width)
Time (min)	5–10 (manual)	20–40 (computer)	1–2 (manual) 1–14 (computer)	Less than 1 (computer)	1–20
Data size	50–70 images	One scene	Per plant	All the data	Per plant
URL	http://www.usa.canon.com; http://www.maxmax.com	http://ccwu.me/vsfm/	http://www.danielgm.net/cc/	https://www.r-project.org/	

### Extraction of leaf midribs with local polynomial function and leaf width with quantile regression

**Fig. 4.**
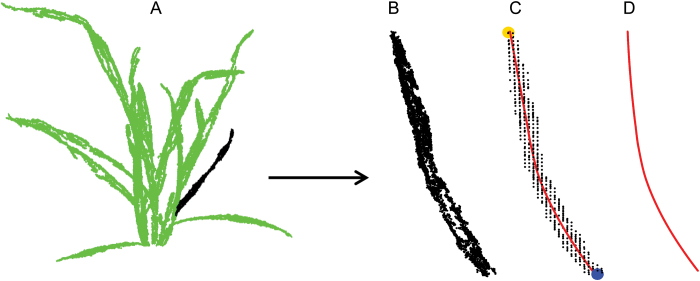
Diagram for the extraction of a leaf structure, 3D tip, and base points. (A) Point cloud of an individual wheat plant, Lincoln, 5 June, 101 363 points, 17 leaves, four tillers. (B) Point cloud of an individual leaf (black points in A). (C) The big circle at the top: tip point; the big circle at the bottom: base point; small points: voxel domain; solid line: fitted structure. (D) The fitted leaf midrib. (This figure is available in colour at *JXB* online.)

At this stage of analysis, we have segmented point clouds of organs for each plant. We fitted the leaf midribs to these segmented point clouds of organs using the local polynomial regression-fitting algorithm (Fig. 4A and B). Points of organs were divided in the 3D voxel domain (Gorte and Pfeifer, 2004) to reduce the point number of organs and speed up calculations. The step size of the voxel domain is the most important parameter and is balanced by the accuracy and performance. In this study, the step size was assigned by one rule, that each voxel included a maximum of 10 points. This reduced run time by 10-fold. All voxels with not more than two points were filtered out as the noise points. The key points of the leaf base and tip were extracted according to the maximum distance among all voxels (Fig. 4C). The key point, which was close to the stem, was assigned as the leaf base (e.g. the big circle at the bottom of Fig. 4C). The leaf base was moved to the origin and the leaf tip was rotated to the positive direction of the *x*-axis. A local polynomial regression-fitting algorithm was used to fit the leaf midribs using the loess function in R programming language (Fig. 4D) (R Development Core Team, 2012). Two values were calculated to estimate maximum leaf width for each point of the leaf: (i) distance to the midrib; and (ii) distance from the leaf base to the corresponding point in the midrib. The leaf edge was identified according to the 90th percentile of all points on either side of the midrib using quantile regression (Koenker, 2015). Quantile regression was used to filter out the noise points around leaf edges which are generated during image reconstruction, either due to lower quality detection or due to small movements of leaves between photos. The maximum leaf width was calculated as twice the maximum value of the estimated leaf edge.

### Extraction of phenotypic attributes

Phenotypic attributes of wheat growth and development were calculated from the segmented organs and the extracted leaf midribs; for example, tiller number, leaf number on each stem, plant height, leaf length and angle, leaf elongation rate, Haun index, and phyllochron. The tiller number and leaf number were directly counted as the number of groups after segmentation. The plant height was estimated as the length between the base point of the plant (soil surface) and the longest leaf tip as if it had been pulled directly vertical (Budak *et al.*, 1995). Leaf length was calculated as the length of the fitted leaf midrib. Leaf angle was defined as the angle between the vector from the base point to the middle point of the leaf midrib and the horizontal plane (Zheng *et al.*, 2008). The elongation rate of leaf length was calculated as the increment of leaf length between two sequential images. The Haun index is calculated following the method of Haun (1973) and is based on the number of expanded leaves and a decimal score approximating the proportion of expanding leaves. Phyllochron was linearly fitted as between the accumulated thermal time after sowing and the Haun index (Slafer and Rawson, 1997). For leaves where leaf width was estimated, leaf area was computed as the product of leaf width and leaf length multiplied by 0.75 (Dodig *et al.*, 2010).

## Results

### Reconstruction of wheat canopies and extraction of leaf midribs

**Fig. 5.**
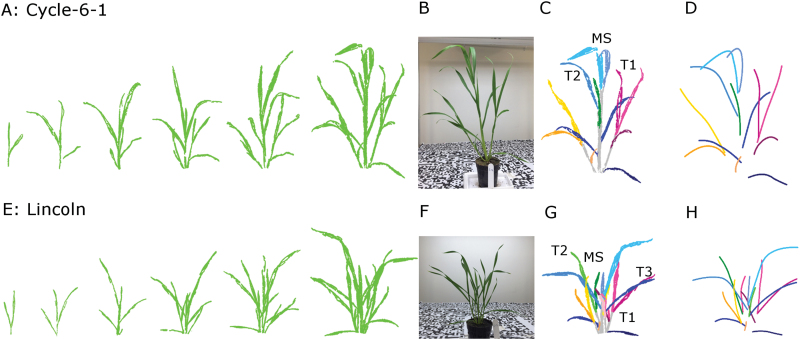
The 3D reconstruction (A and E), the segmentation results (C and G), and leaf midrib extraction (D and H) at the final imaging for Cycle-6-1 and Lincoln. There were 13 imaging dates for Cycle-6-1 and 11 imaging dates for Lincoln, but only six represented stages are shown in this figure. The images in the seventh column were the original photos for Cycle-6-1 (B) and Lincoln (F). For the final photography, Cycle-6-1 had 13 leaves and two primary tillers at the final imaging on 9 June and Lincoln had 17 leaves and three primary tillers at the final imaging on 5 June. All reconstructed canopies were segmented into individual tillers and leaves. The lines were the fitted leaf midribs using a local polynomial regression-fitting algorithm for the structures segmented by leaf rank (Cycle-6-1. D; Lincoln. H). (This figure is available in colour at *JXB* online.)

The point clouds were reconstructed from the multiview image sequence for two genotypes from the first leaf stage of Cycle-6-1 and the second leaf stage of Lincoln to the sixth leaf stage for both lines (Fig. 5A and E). The total point numbers ranged from ~10 000 at the second leaf stage to ~100 000 at the sixth leaf stage for each individual plant. Compared with the photos from the camera, the reconstructed point clouds visually represented canopy structures of wheat seedlings (e.g. Cycle-6-1 and Lincoln at the sixth leaf stage, Fig. 5B and F).

At the final time point, organs were well defined (e.g. Fig. 5C and G for Cycle-6-1 and Lincoln, respectively, at the sixth leaf stage), with the total number of points per organ ranging from 700 to 17 000 depending on organ size. The stems were grouped into main stems and tillers based on the topology of organs [e.g. main stem (MS), T1, T2, and T3 in Fig. 5C and G]. It required the user to take ~1min per leaf to group some of the point clouds when stems or leaves were split. There were 13 and 17 organs in each individual plant for Cycle-6-1 and Lincoln, respectively, and the fitted leaf midribs reflected the 3D structure of organs (Fig. 5D and H for Cycle-6-1 and Lincoln, respectively).

**Fig. 6.**
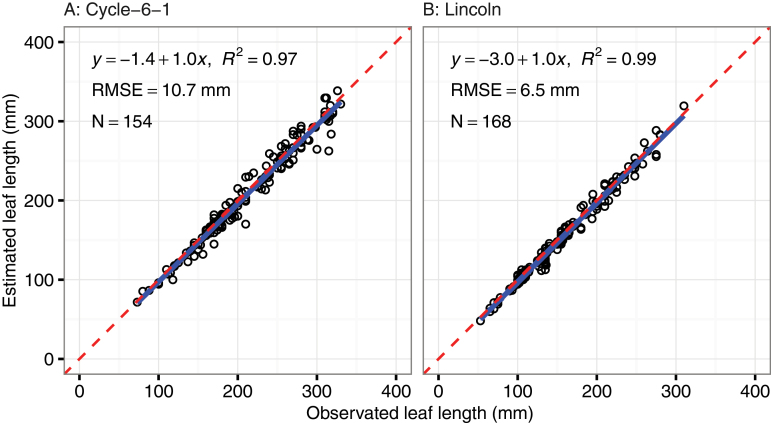
Comparison of leaf lengths between manual measurement and estimation from reconstructed canopies using multiview image sets. (This figure is available in colour at *JXB* online.)

A total of 322 leaf length observations were manually measured for Cycle-6-1 (154 measurements) and Lincoln (168), ranging from 50mm to 340mm. Good agreement was found between measured and estimated lengths (Cycle-6-1, *n*=154, RMSE=10.7mm, *R* ^2^=0.97, *y*= −1.4+1.0*x*; Lincoln, *n*=168, RMSE=6.5mm, *R* ^2^=0.99, *y*= −3.0+1.0*x*; Fig. 6). Estimated leaf widths were also in good agreement with observed values for the key leaves L3 and L4 (*n*=25, RMSE=1.7mm), with slightly larger error for leaves L1 and L2 (*n*=36, RMSE=2.1mm). Relative to the mean measured leaf widths, this relative error was acceptable for L3 [mean measured leaf width for all plants=11.8±1.3mm; mean absolute error=0.9mm (8%)] and L4 [14.4±1.1mm; 1.7mm (12%)], but was relatively large for L1 [6.6±1.5mm; 2.2mm (33%)] and L2 [8.2±1.2mm; 1.6mm (20%)].

### Comparison of phenotypic attributes for two early vigour contrasting genotypes

**Fig. 7.**
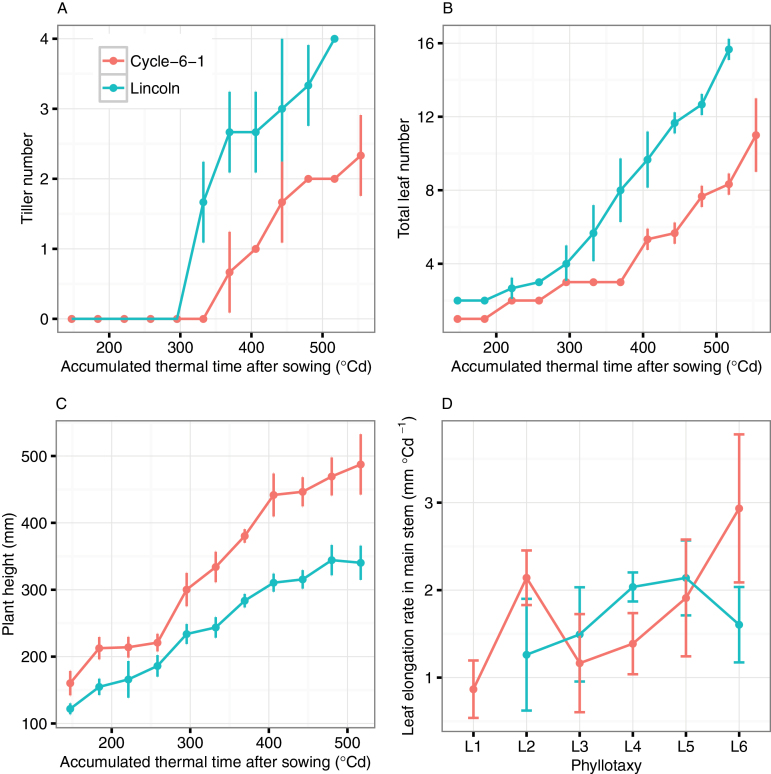
Plant phenotype parameter extraction result for two lines including (A) the dynamic growth of tiller number; (B) the dynamic growth of total leaf number; (C) the dynamic growth of plant height; and (D) leaf growth elongation for different phyllotaxy on the main stem. The error bars indicate the SD from three replicates. (This figure is available in colour at *JXB* online.)

The dynamic evolution of phenotypic attributes of Cycle-6-1 and Lincoln was calculated from the segmented organs and fitted leaf midribs (Fig. 7). By the sixth leaf stage, the tiller number increased to 2.4±0.6 and 4.0±0.0 for Cycle-6-1 and Lincoln, respectively. Lincoln always had one or two more tillers than Cycle-6-1 for the entire seedling stage (Fig. 7A). The total leaf number in the main stem and tillers showed the same trend as the primary tiller number (Fig. 7B; e.g. up to 11.0±2.0 and 15.7±0.6 for Cycle-6-1 and Lincoln, respectively, at the sixth leaf stage). Plant height (from the plant base to the tip of the maximum leaf) increased to 487.4±45.2mm and 340.3±25.6mm for Cycle-6-1 and Lincoln, respectively (Fig. 7C). Although the average elongation rate of leaves on the main stem was similar for the two genotypes (1.7±0.5mm °Cd^−1^ for Cycle-6-1 and 1.7±0.4mm °Cd^−1^ for Lincoln), there was a significant difference in the trend of leaf elongation rate across different leaves (Fig. 7D). The elongation rate of L2 for Cycle-6-1 (2.1±0.3mm °Cd^−1^) was significantly greater than that of L1 (0.9±0.3mm °Cd^−1^, respectively, *P*<0.01). The leaf elongation rate of Lincoln showed a continued growth trend from L2 (1.3±0.6mm °Cd^−1^) to L5 (2.1±0.4mm °Cd^−1^) and then decreased to 1.6±0.4mm °Cd^−1^ for L6.

**Fig. 8.**
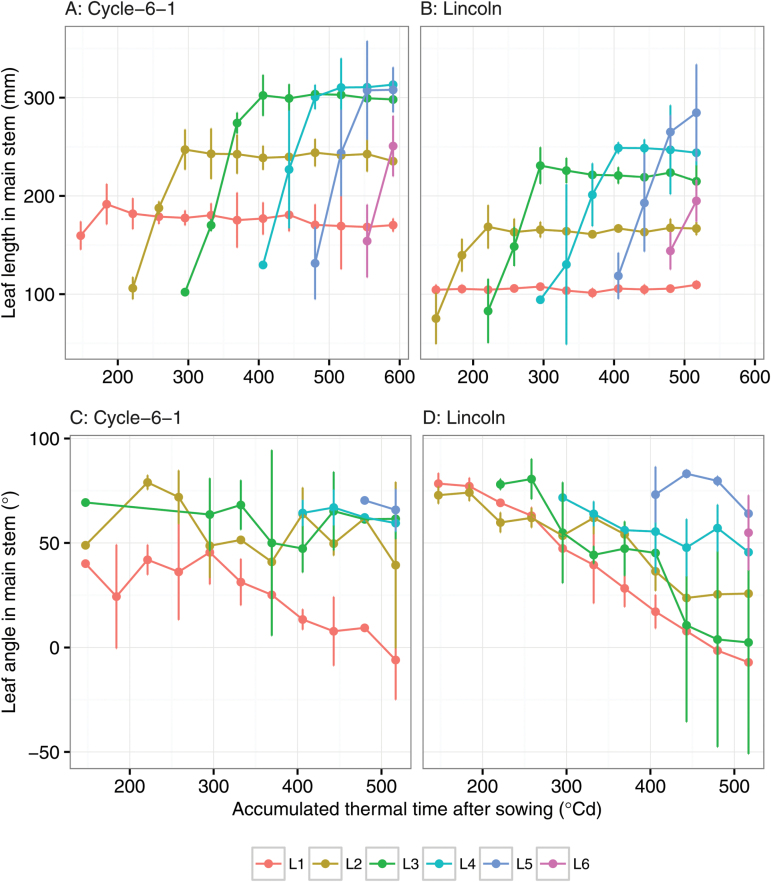
The dynamic growth of leaf length and leaf angle (degrees above horizontal) for leaf rank L1–L6 on the main stem for Cycle-6-1 and Lincoln. The leaf length is estimated from multiview images. The leaf angle is estimated from multiview images and defined as the angle between the vector from the base point to the middle point of the leaf midrib and the horizontal plane. The error bars indicate the standard deviation from three replicates. (This figure is available in colour at *JXB* online.)

Dynamic growth of leaf geometry was obtained from serial photography (e.g. Fig. 8A and B for leaf length and Fig. 8C and D for leaf angle). For the main stem, the final leaf length gradually increased from 105.3±3.8mm for L1 to 284.7±37.7mm for L5 for Lincoln, and from 191.6±16.3mm to 308.0±38.4mm for Cycle-6-1. In general, leaves of Cycle-6-1 were longer than those of Lincoln for each leaf rank on the main stem (Fig. 8A and B). The average estimated leaf width of L3 and L4 was 15.0±2.1mm and 17.1±2.7mm, respectively, for Cycle-6-1, and 8.5±0.9mm and 10.8±1.4mm, respectively, for Lincoln, with significant difference between the two genotypes (*P*<0.01). The measured values of leaf width of L3 and L4 for Cycle-6-1 were 15.6mm and 18.2mm and for Lincoln were 8.1mm and 10.6mm.

The extraction leaf angle (above the horizontal) decreased with the accumulated thermal time for both of the genotypes on all phyllotaxy (L1–L6; Fig. 8C and D), indicating upright leaves at the early stage, then tilting to more horizontal at later stages as the leaves grew longer. This was more notable in Lincoln than in Cycle-6-1, which had less change in angle over time. Comparing leaf angles of the two genotypes, that of Lincoln was 25 ° greater (more erect) than that of Cycle-6-1 at the earlier time of seedling stage and it presented a uniform and stable reduction of ~5–7 ° between two adjacent measurements. Cycle-6-1 showed a much smaller decreasing trend of ~1–3 ° between measurements, especially for the higher phyllotaxy (L3, L4, and L5).

**Fig. 9.**
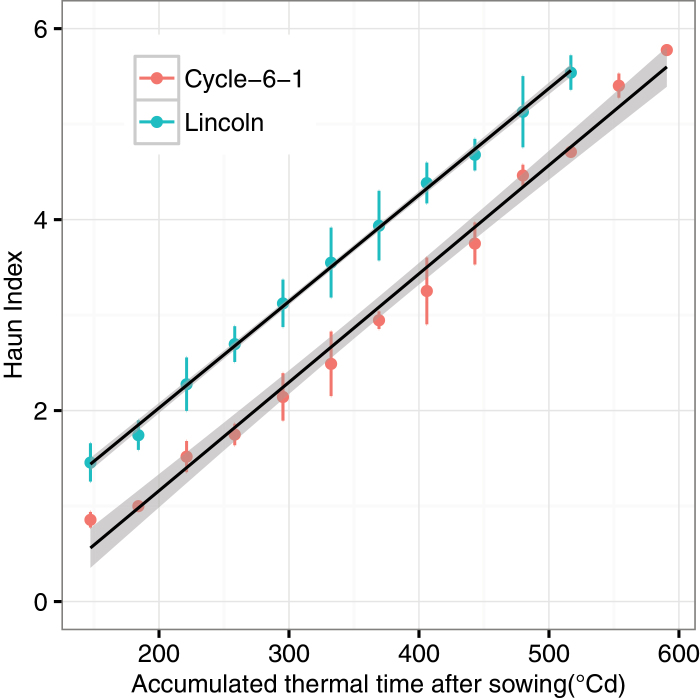
The estimated Haun index for Cycle-6-1 and Lincoln. The black lines are linear regression between the Haun index and accumulated thermal time (*y*= −1.1+0.0011*x*, *R* ^2^=0.99 for Cycle-6-1; *y*= −0.2+0.0011*x*, *R* ^2^=1.00 for Lincoln). The error bars indicate the standard deviation from three replicates. (This figure is available in colour at *JXB* online.)

The Haun index and phyllochron of the two genotypes were calculated using the leaf number and leaf length. Phyllochron was 90.9 °Cd for both of the genotypes (Fig. 9; *R* ^2^=0.99 for Cycle-6-1 and 1.00 for Lincoln). The intercepts of the regression lines, however, were significantly different (−1.1 for Cycle-6-1 and −0.2 for Lincoln, respectively).

## Discussion

### Characterization of crop structure using photography

The point cloud is the basic data set for further extraction of phenotyping attributes (Paproki *et al.*, 2012; Paulus *et al.*, 2013) and can be generated by many technologies, such as a 3D digitizer (Falster and Westoby, 2003; Zheng *et al.*, 2008), LiDAR (Omasa *et al.*, 2007), time-of-flight laser (Kazmi *et al.*, 2014), and ultrasonic sensing (Krammer and Schweinzer, 2006). Compared with other technologies, image acquisition techniques provide an economic, efficient, and convenient method to generate point clouds in a range of plant science applications (Chapman *et al.*, 2014; Lou *et al.*, 2014; Pound *et al.*, 2014). In this study, the point clouds were reconstructed using the VisualSFM system based on the SFM-MVS algorithm from the multiview image set (Fig. 2). Although normal RGB cameras are suitable to phenotype wheat seedlings (data not shown), customized cameras can provide extra benefits through enhancing the contrast of leaves and background, such as the vegetation stress camera in this study or multiple band cameras (MicaSense Red Edge; www.micasense.com). The ‘vegetation stress’ camera is more sensitive to green colour as it has a strong NIR reflectivity. Manual methods involve labour-intensive handling, and destruction of plants for some types of phenotyping. The non-destructive measurement took ~15min to image a single plant (60–70 images). However, an automated camera platform could minimize intervention and provide the capacity for high frequency of sampling (i.e. 3–5 times each day) or lots of treatments (Hartmann *et al.*, 2011).

The extraction of plant phenotypic parameters (e.g. leaf length, angle, and elongation rate) requires segmenting of the reconstructed 3D point cloud into individual organs. In this study, we introduced a semi-automatic method for this step (Fig. 3). Consequently, we can reliably segment wheat seedlings with up to 10–15 leaves per plant and having 2–3 tillers (Fig. 5). The organ segmentation from point clouds becomes difficult for older plants due to occlusion among tillers and leaves. Until now, research on 3D reconstruction from image sets and phenotype parameter extraction of wheat mainly focused on the target of individual plant or canopy scale (Grieder *et al.*, 2015; Vadez *et al.*, 2015). Totally automatic segmentation has only been realized on some broad-leaf plants without tillers or with few leaves, such as *Gossypium* (Paproki *et al.*, 2012) and *Hordeum vulgare* with just three leaves (Dornbusch *et al.*, 2007). Completely automatic segmentation is still a challenge for narrow-leaf crops such as wheat and rice (Wahabzada *et al.*, 2015).

In this study, we mainly focused on extraction of wheat structure related to crop phenology and morphology (Ward *et al.*, 2014). The estimated leaf length from midribs has good agreement with the measurements (RMSE=8.6mm; Fig. 6). The underestimation of leaf length was caused by (i) fewer pixels detected at the small leaf tips which were lost during reconstruction; and (ii) blade bases not clearly segmented from the point cloud. A sensitivity analysis was conducted to check the influence of image number and distribution on 3D reconstruction. The results indicated that more images (>60) did not improve the resolution of image reconstruction, and was sometimes worse. However, the uniform distribution of images in the hemisphere was a key strategy to reconstruct the point cloud.

The maximum leaf width estimates showed reasonable results for larger leaves (e.g. L3 and L4), but were less accurate for smaller leaves (e.g. L1, L2, and new leaves). The higher relative errors for smaller leave appeared to be associated with (i) rolled leaf shape; (ii) lower accuracy of vernier caliper measurement; (iii) the relatively low resolution of the images; and (iv) self-shading by high rank leaves. Algorithms of leaf surface reconstruction have been successfully applied for broad leaves such as cotton, chenopodium (Dorr *et al.*, 2014; Kempthorne *et al.*, 2014), anthurium, and frangipani (Oqielat *et al.*, 2007, 2009). These algorithms, however, are difficult to apply for the surface of thin wheat leaves in the early stage (i.e. leaf width is ~6–15mm up to the sixth leaves; data not shown). Moreover, wheat leaves are twisted in the glasshouse and natural growing conditions, which creates more challenges to fit with simple surface equations. Others have used the discrete smoothing *D* ^2^-spline algorithm to fit the twisted leaf surface of wheat (Kempthorne *et al.*, 2015). However, the point cloud in that work was obtained from a 3D Artec S scanner, which was evenly distributed in 3D space. The point cloud, reconstructed from multiview images, was unevenly distributed, with missing sections caused by self-shading among leaves. A new algorithm is required to build surfaces of thin and twisted leaves, especially from point clouds reconstructed from multiview image sets.

### Dynamic monitoring of growth and development with photography

Vigour in the early stage of growth can increase yield potential and has been selected in breeding, as wheat shows less vigour than other winter cereals (Regan *et al.*, 1992, 1997; Zhang *et al.*, 2015). The traditional phenotyping technologies, however, are time-consuming and destructive due to anthropogenic error and instrument interference (Richards and Lukacs, 2002; Bertin *et al.*, 2010). In recent years, interest in high-throughput phenotyping platforms is increasing in public and private research (Furbank and Tester, 2011; Fiorani and Schurr, 2013). Digital photography provides a promising approach for phenotyping breeding lines with relatively low investment. Data analysis tools enable accurate estimation of ground cover, plant colour, and green biomass at the canopy scale (Casadesús *et al.*, 2007; Mullan and Reynolds, 2010) and for angle, length, and area for individual organs (Clark *et al.*, 2011; Paproki *et al.*, 2012). In this study, the reconstructed point cloud from multiview image sets visually reflected the structures of wheat canopies (Fig. 5A and E). For each time point, the estimated tiller numbers were the same as the measured values based on the integrity of the reconstructed point cloud. The extracted traits [tiller, leaf length, Haun index, etc. (Figs 7–9)] can be used to evaluate phenotypic variation of a breeding population (Rebetzke *et al.*, 2007, 2008; Vos *et al.*, 2010; Dreccer *et al.*, 2013). Further, the derived parameters can be used to run simulation models that could be used to estimate the canopy growth and development in different climatic environments (Chapman *et al.*, 2012; Lobell *et al.*, 2015; Zhang *et al.*, 2016). With the advances of photogrammetry and image processing techniques, automated high-throughput phenotyping shows great promise in plant breeding and agriculture (An *et al.*, 2016). The non-automation part of the workflow can be further improved if new aggregation decision rules can be found.

### Application of phenotyping in breeding programmes and models

Early vigour is the fast development of leaf area at early stages (Rebetzke and Richards, 1999; Maydup *et al.*, 2012). The trait can be achieved through a bigger tiller and leaf number, larger leaf size (Regan *et al.*, 1992), and a vigorous root system (Sprigg *et al.*, 2014). Other traits could be changed to co-ordinate with the vigorous growth of leaves. Phyllochron interval is significantly correlated with length of leaves (Rebetzke and Richards, 1999) as longer leaves tend to take longer to emerge. However, with the method described here, it may be possible to identify lines with a faster elongation rate and or shorter phyllochron and which have early vigour through a different mechanism. In general, the early vigour genotype Cycle-6-1 had the same phyllochron as the non-vigour genotype Lincoln (Fig. 9). However, the Haun index of Lincoln was one unit faster than that of Cycle-6-1 (Fig. 9), which was caused by the longer length of the first, second, and third leaves of the early vigour genotype (1.7-, 1.5-, and 1.4-fold difference in leaf length between Cycle-6-1 and Lincoln; Fig. 8A and B); that is, the appearance rates of the first three leaves were much slower for Cycle-6-1 and this variation of leaf appearance and large leaves may have influenced carbon demand and partitioning to suppress tiller production, as seen in sorghum (van Oosterom *et al.*, 2011). Indeed, the larger leaves of Cycle-6-1 were associated with fewer tillers (Fig. 7). Either combination of fewer tillers with larger leaves or greater tiller number with smaller leaves can increase early vigour to achieve higher biomass (Regan *et al.*, 1992). The estimated maximum leaf width for the third leaf for the early vigour cultivar was almost twice the width of that of the non-vigour cultivar, with the estimated area of leaf three being 3350mm^2^ in Cycle-6-1, much larger than the value of 1420mm^2^ for Lincoln. The width of leaf three had been used in the breeding programme to select advanced lines (Zhang *et al.*, 2015) and can be easily estimated from the photography method when the third leaf has appeared.

The complex interaction of early vigour (G), environment (E), and management (M) requires a selection-specific trait in the target population environment (Cooper and Hammer, 1996; Wilson *et al.*, 2015). Crop models can be useful tools to evaluate the G×E×M interaction in multiple scenarios (Asseng *et al.*, 2003; Chapman *et al.*, 2012; Zheng *et al.*, 2013; Hammer *et al.*, 2014), including future climates (Lobell *et al.*, 2015). Current crop models, however, need to be modified to reflect new knowledge about early vigour (Asseng *et al.*, 2016), such as variation of the Haun index (Fig. 9) and leaf area development. High-throughput phenotyping provides a new capability to assess large numbers of genotypes in a short period (Montes *et al.*, 2007; Cabrera-Bosquet *et al.*, 2012; Araus and Cairns, 2014). Data from the method provided here can be used to parameterize a crop model to improve prediction of the impact of early vigour traits on seasonal growth, water use, biomass, and yield.
